# Strengthening Preventive Care for Culturally and Linguistically Diverse People in Australia: The Case for Community Health Worker Programs

**DOI:** 10.1002/hpja.70226

**Published:** 2026-07-31

**Authors:** Sabuj Kanti Mistry, Uday Narayan Yadav, Soumyadeep Bhaumik, Zohra S. Lassi, Mark Harris

**Affiliations:** ^1^ School of Population Health University of New South Wales Sydney Australia; ^2^ National Centre for Epidemiology and Population Health The Australian National University Canberra Australia; ^3^ International Centre for Future Health Systems University of New South Wales Sydney Australia; ^4^ Meta‐Research and Evidence Synthesis Unit, Health Systems Science George Institute for Global Health Sydney Australia; ^5^ Faculty of Medicine and Health University of New South Wales Sydney Australia; ^6^ George Institute for Global Health New Delhi India; ^7^ School of Public Health, Faculty of Health and Medical Sciences The University of Adelaide Adelaide Australia; ^8^ Robinson Research Institute University of Adelaide Adelaide Australia

## Abstract

Australia is a multicultural country with half of its population from culturally and linguistically diverse (CALD) communities, who experience a disproportionately higher burden of chronic diseases. Yet, people from CALD communities continue to face significant barriers to accessing health and social care services that are essential for effective prevention of chronic diseases. In Australia, prevention is not happening in primary care, with a shortage of workforce and the public health system is underinvested in preventive strategies. In these realities, we highlight that community health workers can play a pivotal role in delivering culturally tailored preventive care in primary care and community settings for CALD communities in Australia.

## 1 Introduction

About half of Australia's population is from culturally and linguistically diverse (CALD) communities with 48.2% being either born overseas or having at least one parent born overseas, and nearly 23% speaking a language other than English at home [1]. Recent analysis shows that total net positive overseas migration in Australia will rise from 9.2 to 14.3 million between 2022 and 2071 [2]. This growing cultural, social and linguistic diversity highlights the need for health systems to address the distinct needs of diverse populations and reduce healthcare disparities. In particular, people from CALD communities experience a disproportionately higher burden of chronic diseases including cardiovascular diseases, diabetes, and kidney diseases [3]. For example, a recent study reports a high prevalence of diabetes (21%), heart disease (12%), and depression (22%) in South Asians living in Australia [4]. Likewise, the prevalence of kidney disease was the highest among people from Polynesian countries such as Samoa (1.5%) and Tonga (1.9%) [3]. While many of these issues require preventive action at the population level, targeted approaches delivered within the health system can significantly improve outcomes, particularly for individuals at higher risk [5]. Accordingly, this commentary focuses on the role of culturally responsive, targeted preventive care delivered within primary care settings for CALD communities in Australia.

## 2 Prevention Gap for CALD Communities

Effective chronic disease prevention requires a holistic approach that addresses complex clinical and non‐clinical needs such as housing, employment, legal and self‐management support [6]. However, people from CALD communities face significant barriers, including limited health literacy, language and cultural differences, dissatisfaction with rushed doctor interactions and system‐related concerns, affordability of health services, difference in preventive services compared to their country of origin and lack of culturally appropriate care limiting their access to and utilisation of both clinical and non‐clinical services [7, 8]. Empirical evidence suggests that the process of adapting to a new cultural environment (acculturation) often introduces behavioural risk factors (e.g., poor housing, job insecurity, tobacco and alcohol use, physical inactivity, and suboptimal dietary practice) which disproportionately affect migrants' health [4, 9]. Moreover, efforts are required to increase uptake of early preventive interventions among people from CALD communities that reduce the risk of chronic diseases, such as screening for cancer [10], and COVID‐19 vaccination [11]. While community‐wide, culturally adapted population‐level interventions (e.g., group‐based physical activity programs, community gardens, or health promotion campaigns) play an important role, they may not be able to sufficiently address individual‐level barriers to adopting and sustaining behaviour change. For those at higher risk, collaborative care within a trusted primary health setting can provide personalised tools necessary to navigate these hurdles, ultimately strengthening preventive efforts and ensuring continuity of care.

The National Strategic Framework for Chronic Conditions [12] prioritised preventive interventions to reduce the burden of chronic disease on society as well as the health system. Consistent with the National Preventive Health Strategy (2021–2030) [13] providing culturally appropriate preventive health services should be a priority. This approach addresses not only individual‐level barriers to accessing care but also strengthens broader determinants of health by improving health and social care system engagement and enables the pathway for decreasing the burden of disease and thereby health systems cost in the long term. A systematic review evaluated culturally appropriate interventions in prevention of chronic diseases among CALD communities [14], reporting that these interventions were effective in increasing knowledge related to chronic disease risk factors, increased adherence to screening for chronic diseases, and improved self‐management practices. It is therefore important to develop targeted, culturally appropriate chronic disease prevention programs for CALD population groups within the Australian health landscape. Yet, a critical question remains: where should preventive care take place? Australia is currently grappling with a shortage of general practitioners (GPs) [15] and nurses [16], many of whom are already overwhelmed by the demands of routine clinical care. Although many GPs cater to the needs of particular language and culture groups, their capacity to provide education and navigation support is limited [17]. Overall, public health systems continue to underinvest in preventive strategies in Australia [13].

## 3 Community Health Workers for Culturally Responsive Preventive Care

Community health workers (CHWs), as trusted community members, have been effective in delivering culturally tailored preventive services within primary care and community settings across developed countries [14]. Compared with broader population‐level interventions, CHWs offer distinct advantages through their ability to provide tailored, personalised support, facilitate ongoing engagement with primary care, promote community mobilisation, and address culturally mediated barriers to preventive service uptake. In Australia, CHW activities have been largely focused on providing one‐to‐one care [18]. However, consistent with international CHW models, their role has expanded to include some group‐based education, such as health promotion delivered by bilingual and bicultural health workers. These CHWs are employed across both primary care and community settings, which are closely integrated in the Australian context. For example, CHWs employed by community‐managed organisations (CMOs) and non‐government organisations (NGOs) work within community settings in Australia while also spending time collaborating closely with primary care providers, including general practitioners and community health services.

Aboriginal Health Workers played a vital role in chronic disease prevention and management for Aboriginal and Torres Strait Islander peoples in Australia, especially through health checks [19] and Integrated team care [20]. Drawing on this model, a tailored CHW workforce could play a complementary role in addressing chronic disease risk factors among CALD communities in Australian primary care and community settings. These CHWs could assess individuals' chronic disease risk and deliver tailored health education to improve health knowledge and promote behaviour change, thereby reducing the burden of chronic disease. They would also identify, refer and help individuals with, or at‐risk of chronic diseases, to navigate both the health and social care services. CHW activities may be delivered one‐to‐one or in groups, depending on the type of intervention (prevention level), community preferences, and how programs are designed within the health systems. However, future research to understand feasibility and contextual applicability of one‐to‐one and group‐based delivery models is required. Understanding which format best supports preventive service delivery will be critical to ensuring that services are not only efficient and scalable, but also responsive to the diverse needs and preferences of CALD communities in Australia. As for example, it is likely that migrants coming from cultures which are communal in nature instead of being individualistic might prefer group‐based care.

## 4 Moving From Evidence to Implementation

Developing CHW programs is a health systems and policy priority but research to inform its development is scarce in Australia. Glenton and colleagues provided a set of 10 reflective questions that policy makers and program planners should consider when designing CHW programs [21]. There is also a clear need for sustainable funding mechanisms, as well as structured training and supervision arrangements for this workforce. In the short term, CHWs could be commissioned and funded through Primary Health Networks, as similar programs are currently supported through this mechanism [22]. In the long term, Medicare funding could support this role through practice incentive payments or fee‐for‐service arrangements within care plans, similar to existing models for Aboriginal Health Workers in Aboriginal Community Controlled Health Services. Effective CHW programs rely on a strong commitment to continuing professional training and structured supervision [18, 23]. Such training and supervision could be provided by the host organisation, with approaches varying according to organisational size, resources, and capacity. This is one reason CHWs may be more appropriately employed by CMOs, even when they are physically embedded within primary care settings. However, these implementation considerations represent key priorities for future implementation research.

Numerous CHW pilot programs have been implemented in local Australian contexts, including peer workers for diabetes and mental health. Greater emphasis is now required on embedding these models within routine primary care, underpinned by sustainable funding and governance structures. While this commentary highlights the strategic necessity of developing a CHW workforce, further health policy and systems research is needed to define the operational frameworks, implementation strategies, and funding models required to effectively develop, integrate, and scale CHW programs in primary care and community settings.

## 5 Conclusion

Chronic diseases pose major challenges for health services and burden in the community. It is therefore important to accelerate efforts at prevention and early detection for chronic diseases. CHWs have the potential to play a pivotal role in improving access to and uptake of preventive care services tailored for specific CALD population groups. However, evidence to guide the optimal design, implementation, and integration of CHW models within the Australian healthcare system remains limited. Moving beyond isolated, short‐term pilot initiatives, there is a need for coordinated investment in the development, rigorous evaluation, and sustainable integration of CHW models. Accelerating this agenda will generate the evidence needed to inform policy, support scale‐up, and reduce persistent inequities in the burden of chronic disease.

## Funding

The authors have nothing to report.

## Conflicts of Interest

The authors declare no conflicts of interest.

## Data Availability

Data sharing not applicable to this article as no datasets were generated or analysed during the current study.
